# Assessing Personality Disorders of People Who Abuse Family Members

**DOI:** 10.1192/j.eurpsy.2024.1264

**Published:** 2024-08-27

**Authors:** A. Mihai, B. Otilia, M. Dan Valeriu Nicolae

**Affiliations:** ^1^Psychiatry; ^2^Paediatric Psychiatry, Mureș County Hospital, Targu-Mures , Romania

## Abstract

**Introduction:**

*Men’s violence against women continues to be a major public health problem worldwide. The long-term consequences require a proper management of resources and a thorough screening protocol. The most extensive study on domestic violence was published in 2005 by the World Health Organization (WHO) and has been updated regularly ever since.*

**Objectives:**

*The aim of this study was to outline a personality profile for people who could be considered domestic abusers and to provide statistical data on personality disorders which are most common among this group of population.*

**Methods:**

*The quantitative data was collected by administering two scales SCID II and Karolinska Scale.*

*Inclusion criteria: People who are physically aggressive with family members.*

*Exclusion criteria: people who are diagnosed with psychosis, people who show aggression with people other than family members*

**Results:**

*
We included 70 people who admit to having committed acts of l physical aggression directed towards family members, who agreed to take part in the study. The scales which were applied are Karolinska scale and SCID II. We identified, using SCID II, DSM IV TR and ICD 10 the following personality disorders types in the 70 intrafamilial aggressors - 10% antisocial personality disorder, 27% borderline personality disorder of which 14% with impulsive emotional instability, 3% obsesive-compulsive personality disorder, 1.4% mixed personality disorder anxious and paranoid.*

**Conclusions:**

*Being able to recognise a personality pattern shows great benefits for screening the patients at risk to develop an aggressive behaviour directed towards family member, thus being a great tool in prevention of long-term consequences associated with living in a hostile environment.*

**Disclosure of Interest:**

None Declared

